# Unleashing the Constructive Potential of Emotions: Some Critical Comments on *Risk, Technology and Moral Emotions* by Sabine Roeser

**DOI:** 10.1007/s11948-020-00195-4

**Published:** 2020-02-11

**Authors:** Steffen Steinert

**Affiliations:** Faculty of Technology, Policy and Management, TU Delft, Delft, The Netherlands

In her latest book, *Risk, Technology and Moral Emotions*, Sabine Roeser makes the case that emotions, particularly moral emotions, can and should play a crucial role in dealing with technological risk. She compellingly shows that emotions are essential for the assessment of, reflection on, and decision making about risk. It is an important book because it is a much-needed corrective to the dominant rationalistic streak that takes emotions to be distortions or distractions and therefore need to be kept out of our reflection of risk. Opposing this dominant mindset, Sabine Roeser convincingly argues in a clear style that there are persuasive philosophical and empirical grounds to believe that emotions give us access to moral values. For this reason, neglecting or down-playing emotions is a grave oversight.

As should be clear from my praise, I think that it is an excellent book. However, I would like to give a critical commentary to the book. In the first part, I will address some opacities and problems with the analogy that is used to characterize ethical intuitionism, the philosophical foundation of the book. I will provide some clarification and outline a problem that arises when we follow the analogy between mathematical axioms and basic moral beliefs. The second part of my critical response is an invitation to Sabine Roeser to say more about the cultivation of emotions.

## The Trouble with Analogies

Let me briefly summarize the contributions of the book before I present some concerns that I have regarding the characterization of ethical intuitionism. *Risk, Technology and Moral Emotions* is important because it makes a compelling case that because emotions are based on moral concerns and values, emotions should be a part of both debates and decision-making about technological risks. The first part of the book is about the current state-of-the-art concerning the role of emotions and moral values in the decision-making about risk. Roeser helpfully distinguishes between three approaches to decision making about risk: technocratic approaches, populist approaches, and participatory approaches. The technocratic approach is problematic because it focuses on quantitative methods and neglects emotions and moral values. Although both populist and participatory approaches take emotions into consideration, they are not satisfying. The populist approach does not assign public emotions a constructive role but takes them to be instrumental for the implementation of technology. The participatory approach includes the public but emotions are rarely taken into account. Roeser also looks at the discussion in empirical decision theory on different perceptions of risk of experts and laypeople. Laypeople’s perception of risk frequently includes qualitative aspects of risk that are usually neglected by the quantitative approaches of experts. Based on ethical intuitionism, that holds that there are objective moral truths and we can know these truths via immediate intellectual awareness, Roeser makes the case that laypeople’s perception of risk includes justified ethical intuitions. For this reason, the intuitive risk perception of laypeople should be included in the decision making about risks.

In the second part of the book, Sabine Roeser turns to emotions and advances an argument to the effect that moral emotions should play a crucial role in decision making about risk. Although recent research in risk perception grants an important role to emotions, it mostly views them as distracting, misleading, or biased. Because of this negative stance towards emotions, even approaches that are sympathetic to the inclusion of laypeople in the decision making about risk resort to more formal and allegedly rational methods. Sabine Roeser provides a diagnosis for this skepticism regarding emotions. The reason is that recent risk perception research subscribes to the dual process model of cognition. According to this model, thoughts and judgements arise from two different systems or processes. One system is said to be automatic, fast, emotional and subconscious, whereas the other system is slow, deliberate and conscious. Sabine Roeser convincingly shows that the dual process model falls short of explaining the risk perceptions and emotions of laypeople because moral emotions fall between the two systems. In order to do justice to emotions, Sabine Roeser develops an alternative account of risk emotions. According to this alternative view, moral risk emotions are perceptions of ethical aspects of risk. She shows that emotions are not necessarily irrational but can be a source of moral knowledge. Risk emotions, like fear and indignation, can direct our attention to moral values such as fairness or justice.

Based on the novel account of risk emotions developed in the second part of the book, the third part of the book develops a procedural approach that includes emotions in the decision making about technological risk. Sabine Roeser shows that although emotions can be distorting, they can also direct our attention to moral concerns. Further, she defends the idea that emotions are critical tools in the reflection on risk because they are helpful in reflecting on both reason and other emotions. The novel approach to emotions is put to practical use in a new participatory approach. The proposed participatory approach supplements existing participatory models because it assigns emotions a more prominent role and includes the emotions of all stakeholders, laypeople and experts. Further, Sabine Roeser shows the fruitfulness of this approach to risk emotions by teasing out the contributions of the account for debates regarding nuclear energy, climate change, public health risks and architecture. Incorporating emotions of the public and experts into deliberation about risk can enhance the dialogue and makes for more inclusive public deliberation.

The philosophical foundation of the book is ethical intuitionism (see chapter 3.4, pp. 40ff.). Broadly speaking, proponents of ethical intuitionism believe that people have an intuitive awareness, or a direct perception, of moral values. These intuitions are said to form the basis of our moral knowledge and our moral reasoning proceeds from this basis (see Audi 2005; Dancy 2014; Huemer 2008). According to Sabine Roeser, and other intuitionists, our ethical intuitions can be understood in analogy to axioms in mathematics: “Ethical intuitionists believe that ethical intuitions function in a similar way as axioms in mathematics in that we cannot argue for them any further, but they can still be taken to be justified” (p. 40).

Unfortunately, the passages in the book about the analogy between ethical intuition and mathematical axioms are not as clear as one wishes. This is mainly due to the fact that Sabine Roeser identifies basic moral beliefs with ethical intuitions. Accordingly, sometimes ethical intuitions are taken to be analogous to axioms, sometimes it seems that she takes basic moral beliefs to be analogous to axioms. However, I don’t think the two are identical and it is more fitting to say that basic moral beliefs are like axioms. I will elaborate why this is the case by first saying something about justification and forms of evidence for axioms.

Axioms and their justification are contested issues in mathematics. The traditional Euclidean conception of justification in mathematics takes axioms to be self-evident and fundamental. However, philosophers and mathematicians have contested this view. For example, Gödel (1947), while proposing mathematical intuition as intrinsic evidence for an axiom (axioms ‘force themselves upon us as being true’), he has also emphasized extrinsic evidence in favor of mathematical axioms. Examples of extrinsic evidence are fruitfulness in consequences or the systematization and uniformization of proofs in second order arithmetic. Russell (1973) took aim at the idea that axioms are self-evident. According to Russell, mathematical axioms are justified to the degree that they are able to explain, entail or systematize other more apparent mathematical propositions. Similarly, Maddy (1988, p. 482) distinguishes between intrinsic and extrinsic justifications of axioms. Maddy specifies the first as obvious and self-evident, whereas the second refers to pragmatic and heuristic justifications such as consequences and explanatory power. Further, she notes that there is a historic process of how maxims become self-evident: “… that from the very beginning, the process of adopting set-theoretic axioms has not been a simple matter of noting down the obvious. Rather, the axioms we now hold to be self-evident were first justified by reference to vague rules of thumb and purely extrinsic consequences, in addition to intrinsic evidence (Maddy 1988, p. 490). More recently, Maddy has argued that the axioms of set theory, which is a branch of mathematical logic, are justified because they enable set theory to unify mathematics (Maddy 2011). The justification of the axioms of set theory is extrinsic, in that “all that really matters is the fruitfulness and promise of the mathematics itself” (Maddy 2011, p. 117).

Based on Maddy, Gödel and Russell we may say that there are two kinds of evidence when it comes to mathematical axioms: (1) intrinsic evidence, that is mathematical intuition, and (2) extrinsic evidence, like explanatory power and fruitfulness. As mentioned above, Sabine Roeser identifies basic moral belief and intuition. I find that somewhat puzzling in the light of the proposed analogy to axioms. The analogy says that ethical intuitions are like axioms and that means that ethical intuitions have intrinsic and extrinsic evidence. The puzzling implication is that the intrinsic evidence for the axiom/ethical intuition, is, by analogy, another ethical intuition. I think that the more fitting analogy is between mathematical axioms and basic moral beliefs. In this analogy, both axioms and basic moral beliefs are backed by mathematical and ethical intuition, respectively. This is also in line with passages in the book that treat basic moral beliefs as basis for more complex moral beliefs. For instance, on page 40 of the book, Sabine Roeser says that “our complex ethical beliefs are based on basic moral beliefs or intuitions”. Similar to complex theories that are based on axioms, complex moral beliefs can be based upon basic moral beliefs. In a nutshell the clarified picture looks like this: By analogy to axioms our basic moral beliefs can have (1) intrinsic evidence, in the form of ethical intuitions, and (2) extrinsic evidence. Extrinsic evidence could be something like fit with other basic moral beliefs. Further, these basic moral beliefs can form the basis of complex moral beliefs. I will proceed under the assumption that this clarified picture is correct.

Sabine Roeser argues that we should understand ethical intuitions as moral emotions (e.g., pp. 91 ff.). Based on what I have outlined above regarding the analogous relation between axioms, ethical intuitions and basic moral beliefs, I take this to mean that moral emotions serve as intrinsic evidence for basic moral belief, not that moral emotions are basic moral beliefs. Emotion plays the same role for basic moral beliefs as mathematical intuition plays for axioms. The question I kept asking myself while reading the passages in the book about moral emotions and intuitions was this: Do we have access to basic moral beliefs (or values) *exclusively* via moral emotions or are there other forms of ethical intuition that are non-emotional? Because if there are other kinds of ethical intuitions for basic moral beliefs, one may wonder why we should privilege the form of ethical intuition that takes the form of moral emotion. Then again, for the sake of parsimony we may want to assume that there really is only one kind of ethical intuition. For axioms there is just one kind of intuition at work and this intuition is rational and non-emotional. For instance, Parsons (2000) puts forth an account of mathematical intuition as rational intuition that is not like a perception of objects. Sabine Roeser, like other intuitionists, takes ethical intuition to be similar to perception. Consider the following quote from the book (p. 5): “Ethical intuitionism is a philosophical approach that takes intuitions as prima facie reasonable and justified direct perceptions of objective moral values”. To summarize, it seems that the analogy between basic moral beliefs and axioms nudges us to think that intuition for basic moral beliefs only takes one form. Basic moral beliefs are supposed to be analogous to axioms and axioms only have one form of intrinsic evidence and that is rational non-emotional intuition.

I think the issue of whether there are multiple forms of intuition that serve as intrinsic evidence for basic moral beliefs matters for Sabine Roeser’s project. If she admits that there is another form of ethical intuition than moral emotions, then I am really interested to hear about the differences and similarities between the two. Specifically, I would like to hear whether one form gives us better access to basic moral beliefs or moral values than the other form. Sabine Roeser argues that we should give emotions a more prominent role, for example in deliberation. If it turns out that emotions are not the only form of intuition and that another form of moral intuition is also suited to give us access to moral value and basic moral beliefs, it is not clear whether and why we should give emotions a more prominent role. It is my hope that Sabine Roeser can strengthen her account by addressing the issue of multiple intuitions I have just sketched.

## Going Forward: Unleashing the Constructive Potential of Emotions

I will now turn to a topic that I think is very important but absent in the book. This topic is the cultivation of emotions and I am very eager to hear Sabine Roeser’s thoughts about it.

Emotions give us access to our values and moral concerns. Here is an exemplary quote from the book: “Emotions can provide important moral insights via care, sympathy, empathy and compassion, and feelings of responsibility, justice and indignation. Emotions help us to reflect on the values that we find important and how our actions relate to our lives and those of others” (p. 131). Given that emotions are so important, it seems to be a good idea to cultivate and refine them. And indeed, throughout the book it is mentioned, or sometimes hinted at, that emotions can be influenced and that they should be cultivated and educated (e.g., pp. 129 ff.). It seems that Sabine Roeser is suggesting that the refinement of emotions, for example compassion and feelings of responsibility, will improve our access to basic moral beliefs. Further, I understand the sections about emotions and democratic decision making (e.g., ch. 7.2) as a suggestion that attending to emotions will improve citizenship and the democratic processes. For instance, it is proposed that emotions can provide us with a better understanding of other people and their perspective. Also, emotions like compassion and feelings of justice provide moral insights into our own moral concerns. Understanding others and understanding our own values is something that we deem important for democratic decision making. This gives us reason to hone our abilities to understand other people’s perspective and our own moral values.

Experience seems to show that mathematical intuition can be refined and Kurt Gödel argued that we can cultivate mathematical intuition (see Tieszen 2002). Based on the above-mentioned analogy between moral intuition, that is moral emotions, and mathematical intuition, one could assume that emotions can be cultivated like mathematical intuition. There is another analogy in the book to characterize moral intuitions that more strongly suggests that ethical intuition can be cultivated. Recall that moral intuitions are like sense perception and that moral intuitions are perceptions of moral reality (p. 40). Sense perception can be cultivated because it is hard to deny that through experience people usually develop better abilities for observation. For instance, bird watchers and referees have improved perceptual abilities.

Turning to emotions, it is harder to see how one would exactly go about cultivating emotions. On the one hand, it seems that perceptual processes cannot be easily changed. The stick in the water will look bent no matter how much I train my visual perception. On the other hand, there are cases that suggest that perception can be cultivated. For example, the music or wine connoisseur has a much higher auditory and gustatory sensitivity than most people. Something like connoisseurship seems to be in line with what Sabine Roeser has in mind when she refers to emotional sensitivity. At one point, she suggests that emotionally sensitive people have a greater ability to make fine-grained distinctions (p. 94). It would have been interesting to learn more about how she thinks emotions can be transformed or cultivated, given that it seems important to do so. Alas, she does not go into any specifics in this regard but only leaves a few remarks here and there. For example, she mentions that ethical intuitions can be the result of a long process of reflection (p. 81), which is vaguely suggestive of a way of cultivating ethical intuitions.

In the book, emotions are described as a source of wisdom (e.g., p. 97) and it is argued that emotions like empathy can help us find out what is important and can also provide us with a better understanding of other people. Although I agree that some emotions can play such a role in some circumstances, they are not always a progressive force or lead to a better understanding of other people. For instance, empathy may not be as conducive to tolerant and peaceful co-existence as it is typically assumed to be. Some authors, like psychologist Bloom (2018), have made the case that empathy is not necessarily a force for good. Empathy can facilitate morally questionable conduct and motivate cruel actions. The same emotion can have different consequences, depending on the circumstances. Also, history provides ample evidence where emotions, particularly collective emotions like collective anger or collective outrage, have fueled hate, exclusion and violence. Some emotions can spur anti-democratic sentiments and emotions like fear are usually a big factor in the rejection of out-groups. Nevertheless, it is plausible that in some circumstances, for instance if they are appropriately channeled, even emotions like collective anger can unfold a predominantly progressive potential.

I would like to suggest that only under certain conditions can emotions play a constructive and progressive role. That is, some emotions *can* be a source of wisdom and emotions *can* have beneficial effects only if they are embedded in a framework that is conductive to this. There needs to be some sort of supportive scaffolding (e.g., institutions, procedures) in place that allows emotions to unfold their positive potential, individually and collectively. Given that Sabine Roeser seems to think that emotional sensitivity is important, I would have liked to hear more about the circumstances and frameworks in which emotions can unleash their constructive potential and where emotions are facilitating democracy. This seems a particularly important issue in an age where emotions in politics seem to play a rather dividing role.

I would like to raise a similar concern regarding the idea that techno-art can play an important role in the deliberation of technology (see pp. 160 ff.). The claim in the book is that art appeals to imagination and compassion. That may be true but does not mean that art makes people more compassionate or that engagement with art makes for better deliberation. It is at least doubtful that an engagement with art actually makes people more compassionate. I could not find any empirical study that shows that this is the case. Sabine Roeser writes that “techno-art can potentially make a constructive contribution to emotional-moral reflection on the risks of technical development” (p. 166). I think the word ‘potentially’ is important here because the constructive power of emotions needs to be unlocked somehow. Whether or not art can make a positive contribution to compassion and deliberation seems to depend on the *kind* of engagement with art. Again, it appears that the environment needs to be conducive, so that the constructive, rather than the destructive, potential of emotions can be harnessed. The interesting question is how exactly such a framework would look like.

If we are serious about emotional cultivation and raising the emotional sensitivity of people, we need to think about practical ways of doing so. Unfortunately, Sabine Roeser is silent on how a framework or scaffolding for unlocking emotion’s potential might look like. She helpfully provides 10 considerations to enhance emotional deliberation (pp. 131ff.). The list includes talking openly about emotions, engaging in a dialogue with all involved people, and a symmetrical discussion between laypeople and experts. However, although I think that these suggestions can facilitate deliberation, they concern a one-off affair and may not easily be translated to a broader scale. I would have really liked to read some practical suggestions about how to go about emotion cultivation and enhancing the emotional sensitivity of people.

One way to foster emotional sensitivity could be the creation of institutions where citizens can hone their emotional skills. Maybe something along the lines of night schools that offer courses on how to improve emotional sensitivity. Another idea is that we make emotional cultivation a part of the regular curriculum. We may want our schools to nurture emotion literacy. Schools and universities could offer classes on how to (critically) engage with emotions or educators could teach compassion (provided that it can be taught, of course). For example, the Yale Center for Emotional Intelligence advocates and supports the teaching of emotional intelligence in schools. Maybe even technology can be helpful in cultivating emotions. Virtual reality (VR) technologies could be used to enhance emotional sensitivity. With the help of VR, people can literally step in another person’s shoes and experience a novel situation. For example, researchers from Stanford University developed a VR scenario where participants experience becoming homeless (Herrera et al. 2018). The researchers say that their results indicate that VR has a long-lasting positive effect on empathy towards homeless people. One could imagine that institutions use VR to offer empathy training sessions.

I would really like to hear Sabine Roeser’s thoughts on the issues of cultivation of emotion and how to enhance emotional sensitivity. In a nutshell, I have three principal questions for her. One is theoretical and the other two are practical: Is emotion the kind of thing that can be cultivated (similar to taste)? (The analogy to mathematical intuition and perception seems to suggest so).; What are ways to cultivate emotions and enhance emotional sensitivity?; What supporting environment or scaffold (e.g., institutions, processes) need to be in place so that emotions can unfold their beneficial potential (e.g., in the service of democracy)?

To wrap up, in this critical response I have addressed and ameliorated some opacities in the book regarding the relation of basic moral beliefs and ethical intuitions vis-à-vis axioms and mathematical intuitions. Based on a clarified picture of this relation I have presented the potential problem that following the analogy between axioms and basic moral beliefs casts some doubt whether we should accept emotions as form of ethical intuition and whether we should privilege emotions over other forms of intuitions. In the last part, I outlined a blind spot of the book, namely the cultivation of emotions.

Again, I would like to stress that *Risk, Technology and Moral Emotions* is a major contribution to the study of how emotions, technology and risk perception relate to each other. I am also aware that no book can address every interesting aspect and my remarks in this critical piece are an invitation to Sabine Roeser to elaborate a little on a worthwhile topic. This, I think, will be in the spirit of her work and may help to extend her ideas into new areas.
